# Variations in pleural microbiota and metabolic phenotype associated with malignant pleural effusion in human lung adenocarcinoma

**DOI:** 10.1111/1759-7714.14988

**Published:** 2023-06-12

**Authors:** DanHui Huang, JinZhong Zhuo, CuiPing Ye, XiaoFang Su, YueHua Chen, Cui Li, LiSan Lin, LaiYu Liu, Haijin Zhao, Tingyue Luo, QianNan Ren, JianHua Wu, Shaoxi Cai, Hangming Dong

**Affiliations:** ^1^ Chronic Airways Diseases Laboratory, Department of Respiratory and Critical Care Medicine Nanfang Hospital, Southern Medical University Guangzhou China; ^2^ Department of Radiation Oncology, Nanfang Hospital Southern Medical University Guangzhou China; ^3^ Department of Oncology Nanfang Hospital, Southern Medical University Guangzhou China

**Keywords:** carboxylic acids, fatty acids, glycerophospholipids, malignant pleural effusion, metabolome, microbiome

## Abstract

**Background:**

Lung cancer is the most common cancer‐related death worldwide. In 2022, the number of daily deaths of lung cancer was estimated to reach around 350 in the United States. Lung adenocarcinoma is the main subtype of lung cancer and patients with malignant pleural effusion (MPE) suffer from poor prognosis. Microbiota and its metabolites are associated with cancer progression. However, the effect of pleural microbiota on pleural metabolic profile of MPE in lung adenocarcinoma patients remains largely unknown.

**Methods:**

Pleural effusion samples collected from lung adenocarcinoma patients with MPE (*n* = 14) and tuberculosis pleurisy patients with benign pleural effusion (BPE group, *n* = 10) were subjected to microbiome (16S rRNA gene sequencing) and metabolome (liquid chromatography tandem mass spectrometry [LC‐MS/MS]) analyses. The datasets were analyzed individually and integrated for combined analysis using various bioinformatic approaches.

**Results:**

The metabolic profile of MPE in lung adenocarcinoma patients were clearly distinguished from BPE with 121 differential metabolites across six significantly enriched pathways identified. Glycerophospholipids, fatty and carboxylic acids, and derivatives were the most common differential metabolites. Sequencing of microbial data revealed nine significantly enriched genera (i.e., *Staphylococcus*, *Streptococcus*, *Lactobacillus*) and 26 enriched ASVs (i.e., species *Lactobacillus_delbrueckii*) in MPE. Integrated analysis correlated MPE‐associated microbes with metabolites, such as phosphatidylcholine and metabolites involved in the citrate cycle pathway.

**Conclusion:**

Our results provide substantial evidence of a novel interplay between the pleural microbiota and metabolome, which was drastically perturbed in MPE in lung adenocarcinoma patients. Microbe‐associated metabolites can be used for further therapeutic explorations.

## Graphical abstract

Pleural effusion samples collected from MPE patients (*n* = 14) and tuberculosis patients with benign pleural effusion (BPE group, *n* = 10) were subjected to microbiome (16S rRNA gene sequencing) and metabolome (liquid chromatography tandem mass spectrometry, LC‐MS/MS) analyses. Our results provide substantial evidence of a novel interplay between the pleural microbiota and metabolome, which was drastically perturbed in MPE. Microbe‐associated metabolites can be used for further therapeutic explorations.

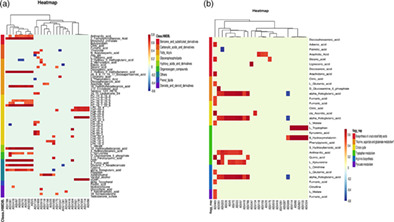

## INTRODUCTION

Lung cancer is the most common cancer‐related death worldwide. In 2022, the number of daily deaths of lung cancer was estimated to reach around 350 in the United States.^1^ Among them, lung adenocarcinoma was the main histological subtype. Malignant pleural effusion (MPE), a special lung cancer status that indicates that the tumor has metastasized or spread to the pleural cavity, is a hallmark of the M1a stage. Although there are various treatment options, patients with MPE suffer from a poor prognosis with a median survival time of 4–11.5 months and a 5‐year survival rate of 3%–10%.^2^, ^3^

Metabolites are small molecules that reflect physiological phenotype of disease. MPE of lung cancer is characterized by a wide variety of cellular and molecular activities resulting in a broad spectrum of possible metabolic products. Dysregulated metabolic behaviors such as unique lipid and amino acid metabolism have been evidenced in MPE caused by lung cancer,^4^, ^5^, ^6^ revealing the clinical utility of metabolic profiles to accurate diagnosis and develop potential target therapy for lung cancer patients with MPE. Apart from host metabolism, complex interaction between microbiota and lung cancer might be crucial to the development of lung cancer.^7^ Emerging evidence has related lung cancer to the composition and function of microbiota in airway, gastrointestinal tract and tumor tissue.^7^ Recent evidence suggested that microbiota may exist in pleural effusion. However, the relationship between pleural fluid microbiome and MPE remains in its infancy. A pioneered study using 16S rRNA sequencing demonstrated the existence of pleural fluid microbiome by excluding potential reagent contamination and possible skin contamination during thoracentesis.^8^ In addition, the study reported unique microbiota associated with MPE caused by lung or breast cancer.^8^ In detail, compared with benign pleural effusion, microbiota in MPE showed greater α diversity and was enriched with genera *Allobaculum*, *Stenotrophomonas*, and *Epulopiscium*.^8^ It is now obvious that some important factors that associate microbiota and cancer are microbial metabolites.^9^ However, the metabolic function of microbiota in MPE remains unknown. Regarding the relationship of microbiota with the host metabolic phenotype, it is very important to assess the pleural fluid microbiota‐related metabolic phenotype alterations, which would help further understand the pathophysiological mechanism of MPE and explore the role of pleural microbiota in the development of MPE.

In this study, we hypothesized that pleural metabolome, linked to MPE of lung cancer, may be in part microbially derived or modified. A case–control study was conducted to link information on pleural effusion microbiota and pleural metabolite data by using 16S rRNA sequencing and liquid chromatography–tandem mass spectrometry (LC–MS/MS) based global metabolomic. Our integrative analyses aimed to identify unique metabolic profile and differential microbial signatures of MPE and to decipher the potential microbial metabolites that were associated with MPE.

## METHODS

### Patients and controls

Both MPE and tuberculous pleural effusion are common etiologies in China. Compared with pneumoniae‐associated pleural effusion, autoimmune disease‐associated pleural and transudative pleural effusion, patients with tuberculous pleural effusion frequently have similar pleural fluid profiles with MPE.^10^ In addition, pneumoniae‐associated, autoimmune disease associated pleural effusion patients may have a unique pleural microbiome profile.^11^, ^12^ Therefore, we only chose tuberculosis pleurisy as the control group in this study. A total of 14 lung adenocarcinoma patients and 10 tuberculosis patients from the Department of Respiratory and Critical Care, Nanfang Hospital, Southern Medical University, Guangzhou, Guangdong, China were included from November 2019 to September 2021. The inclusion criteria were as follows: (1) Diagnosis of malignant pleural effusion was confirmed by positive pleural fluid cytology or pleural biopsy; tuberculosis pleurisy was confirmed with clear microbiological, histopathological or genetic evidence for *Mycobacterium tuberculosis* infection or met the clinical diagnostic criteria. Clinical diagnostic criteria of tuberculous pleural effusion were defined as lymphocyte dominant exudate in the pleural fluid based on Light's criteria, with ADA >20 IU/L, and clinical improvement after antituberculosis medication;^13^ (2) aged 18–79 years old; (3) patients had not received any kind of antitumor therapy (surgery, chemotherapy, immune therapy, targeted therapy) and antituberculosis medication; (4) patients without other malignant diseases and severe heart, kidney, liver dysfunction and without certain respiratory diseases include community acquired pneumonia, acute bronchitis, acute exacerbation of chronic obstructive pulmonary disease, bronchiectasis with infection or asthma. The exclusion criteria were as follows: (1) Patients without a confirmed diagnosis or did not meet criteria of probable tuberculosis pleurisy; (2) aged ≤17 or ≥80; (3) the lung cancer patients had received surgery, radiotherapy or systemic therapy before sample collection; the tuberculosis patients had received antituberculosis therapy before sample collection; (4) patients with any kind of respiratory diseases included community acquired pneumonia, acute bronchitis, acute exacerbation of chronic obstructive pulmonary disease, bronchiectasis with infection or asthma; or with prior history of other malignant diseases; or with serious heart, kidney, liver dysfunction.

### Sample collection and storage

A sample was collected immediately after the first thoracentesis of the patient with pleural effusion. A 15 mL pleural effusion sample was collected and placed in a sterile test tube and stored in a refrigerator at −20°C, and then stored in a refrigerator at −80°C (within 1 week) until delivery to the laboratory for processing and analysis.

### Pleural effusion microbiota analysis

Pleural effusion microbiota analysis was performed by polymerase chain reaction (PCR) amplification.

#### Extraction of genome DNA

The CTAB method was used to extract the total genome DNA in samples. DNA concentration and purity were monitored on 1% agarose gels. According to the concentration, DNA was diluted to 1 ng/μL with sterile water.

#### Amplicon generation

16S rRNA genes in V3–V4 regions were amplified with specific primer (16S_341F:5'‐CCTAYGGGRBGCASCAG‐3';16S_806R:5‐GGACTACNNGGGTATCTAAT) and barcodes. All PCR mixtures contained 15 μL of Phusion High‐Fidelity PCR Master Mix (New England Biolabs), 0.2 μM of each primer and 10 ng target DNA, and cycling conditions consisted of a first denaturation step at 98°C for 1 min, followed by 30 cycles at 98°C (10 s), 50°C (30 s) and 72°C (30 s) and a final 5 min extension at 72°C.

#### PCR products quantification and qualification

We mixed an equal volume of 1x loading buffer (contained SYB green) with PCR products and performed electrophoresis on 2% agarose gel for DNA detection. The PCR products were mixed in equal proportions, and then Qiagen gel extraction kit (Qiagen) was used to purify the mixed PCR products.

#### Library preparation and sequencing

Following the manufacturer's recommendations, sequencing libraries were generated with NEBNext Ultra IIDNA Library Prep Kit (cat no. E7645). The library quality was evaluated on the Qubit 2.0 fluorometer (Thermo Scientific) and Agilent Bioanalyzer 2100 system. Finally, the library was sequenced on an Illumina NovaSeq platform and 250 bp paired‐end reads were generated.

#### Microbiota analysis

Raw data were obtained and then further filtered to eliminate reads with adapter pollution and low quality to obtain an amplicon sequence variant (ASV) table by using QIIME2.^14^ The sequences were thereby denoised by using DADA2^15^ which includes a strict quality control by discarding reads with ambiguous bases, singletons and chimera. ASVs were taxonomically classified with the SILVA database 138 by using the naïve Bayesian algorithm provided in QIIME2.

We applied ASV data in online microbiome data analyze platform (MicrobiomeAnalyst) (https://www.microbiomeanalyst.ca/) to compare microbiota community structure at both intercommunity and α‐diversity and β‐diversity levels. For α diversity, we chose Chao1 value, and Simpson and Shannon indexes for evaluation. For β diversity, we estimated using unweighted distance and visualized by principal coordinate analysis (PCoA). Differential taxonomy was identified by linear discriminant analysis effect size (LEfSe) analysis in an online platform (GALAXY) (http://huttenhower.sph.harvard.edu/galaxy). PICRUSt2^16^ was used to predict the functional profiling of microbial communities based on the 16S rRNA sequences. The network analysis was carried out with SparCC.^17^ The *p*‐value ≤0.05 and SparCC correlation scores ≥0.5 or ≤−0.5 were included for network inference.

### Untargeted metabolomic analysis

#### Metabolite extraction

The samples (100 μL) were placed in Eppendorf (EP) tubes and resuspended with prechilled 80% methanol by well vortex. Then the samples were incubated on ice for 5 min and centrifuged at 15 000 *g*, 4°C for 20 min. Some of the supernatant was diluted to final concentration containing 53% methanol by LC‐MS grade water. Samples were subsequently transferred to a fresh EP tube and were then centrifuged at 15000 *g*, 4°C for 20 min. Finally, the supernatant was injected into the LC‐MS/MS system analysis.^18^, ^19^

#### LC/MS analysis

LC‐MS/MS analyses were performed using a Vanquish UHPLC system (ThermoFisher) coupled with an Orbitrap Q Exactive HF mass spectrometer (Thermo Fisher) in Novogene Co., Ltd. Samples were injected onto a Hypesil Goldcolumn (100 × 2.1 mm, 1.9 μm) using a 17‐min linear gradient at a flow rate of 0.2 mL/min. The eluents for the positive polarity mode were eluent A (0.1% FA in water) and eluent B (methanol). The eluents for the negative polarity mode were eluent A (5 mM ammonium acetate, pH 9.0) and eluent B (methanol).The solvent gradient was set as follows: 2% B, 1.5 min; 2%–85% B, 3 min; 100% B, 10 min; 100%–2% B, 10.1 min; 2% B, 12 min. Q Exactive HF mass spectrometer was operated in positive/negative polarity mode with spray voltage of 3.5 kV, capillary temperature of 320°C, sheath gas flow rate of 35 psi and aux gas flow rate of 10 L/min, S‐lens RF level of 60, aux gas heater temperature of 350°C.

#### Data processing and metabolite identification

The raw data files generated by UHPLC‐MS/MS were processed using the Compound Discoverer 3.1 (CD3.1, ThermoFisher) to perform peak alignment, peak picking, and quantitation for each metabolite. The main parameters were set as follows: retention time tolerance, 0.2 min; actual mass tolerance, 5 ppm; signal intensity tolerance, 30%; signal/noise ratio, 3; and minimum intensity. After that, peak intensities were normalized to the total spectral intensity. Normalized data were used to predict the molecular formula based on additive ions, molecular ion peaks and fragment ions. Peaks were then matched with the mzCloud (https://www.mzcloud.org/), mzVault and MassList database to obtain the accurate qualitative and relative quantitative results. Statistical analyses were performed using the statistical software R (R version R‐3.4.3), Python (Python 2.7.6 version), and CentOS (CentOS release 6.6).

#### Metabolomic data analysis

These metabolites were annotated using the KEGG database (https://www.genome.jp/kegg/pathway.html), HMDB database (https://hmdb.ca/metabolites) and LIPID Maps database (http://www.lipidmaps.org/). Orthogonal partial least squares discriminant analysis (OPLS‐DA) was performed. We applied univariate analysis (*t*‐test) to calculate the statistical significance (*p*‐value). Metabolites with VIP >1 and *p*‐value ≤0.05 and fold change ≥2 or ≤0.5 were considered to be differential metabolites. For clustering heat maps, the data were normalized using *z*‐scores of the intensity areas of differential metabolites and were plotted by Pheatmap package in R language.

### Statistical analysis

Continuous variables were compared between two groups by Mann–Whitney U test or independent *t* test. The categorical variables were compared by chi‐square test, continuity‐adjusted chi‐square test, Fisher's exact test. A *p*‐value <0.05 was considered statistically significant. Spearman's analysis was used to explore the correlation between taxonomies and metabolites. Correlation with *p*‐value <0.05 and |r| > 0.5 is shown in heat maps.

## RESULTS

### Baseline characteristics of subjects

In this study, 14 lung adenocarcinoma patients with MPE and 10 tuberculosis patients with benign pleural effusion (BPE) were enrolled. The clinical characteristics of the 24 patients are listed in Table S1. Subjects in the MPE group were older than those in the BPE group. More patients in the BPE group tended to be male and current or past smokers. MPE and BPE subjects were similar with respect to the factors of antibiotics usage within 1 month and BMI.

### Characterization of pleural microbiota associated with MPE in lung adenocarcinoma patients

**FIGURE 1:**
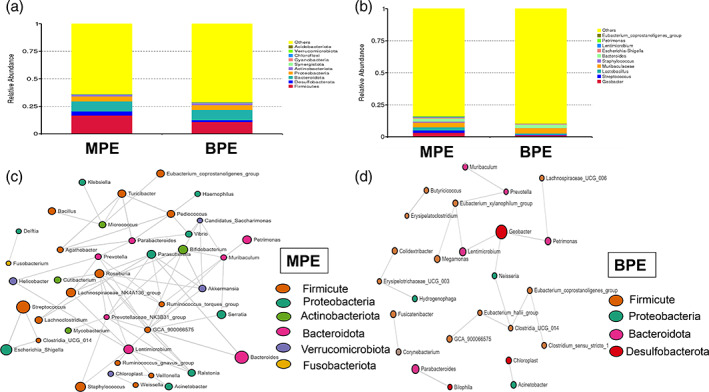
Composition and structure of pleural microbiome community of the malignant pleural effusion (MPE) and benign pleural effusion (BPE) groups. (a)Top 10 phyla of the MPE and BPE groups. (b) Top 10 genera of the MPE and BPE groups. (c) Genera co‐occurrence network based on SparCC of patients in the MPE group. (d) Genera co‐occurrence network based on SparCC of patients in the BPE group.

The average number of trimmed sequences reads number of the 24 subjects was 52 952 (40 380, 94 693). A total number of 9097 ASVs were identified. Rarefaction curve was constructed to evaluate sequence depth (Figure S1). The result indicated that sequence depth of pleural samples was sufficient to reach a reliable estimate of microbiome structure. The top three phyla among MPE and BPE subjects were both phylum *Firmicutes* (16.65% for MPE; 10.78% for BPE), *Bacteroidetes* (9.08% for MPE; 9.38% for BPE), and *Proteobacteria* (4.42% for MPE; 4.51% for BPE). The top 10 phyla of both MPE and BPE are shown in Figure 1a. Among the MPE group, the top five genera were *Muribaculaceae* (3.58%), *Geobacter* (3.25%), *Lactobacillus* (2.64%), *Bacteroides* (2.39%), and *Streptococcus* (1.77%). Among the BPE group, the top five genera were *Muribaculaceae* (4.16%), *Bacteroides* (2.69%), *Geobacter* (1.20%), *Lactobacillus* (1.08%), *Lachnospiraceae_NK4A136_group* (0.9%). The top 10 genera among MPE and BPE were shown in Figure 1b. Coabundance analysis based on SparCC was conducted to analysis the correlation network among the two groups (Figure 1c, d). The pleural microbiota structure of MPE patients was more complex and better organized than the taxonomy structure inferred for patients in the BPE group. The correlation network of MPE group was composed of 42 genera while the structure inferred for the BPE group was composed of 25 genera. The number of intergenus correlations in the MPE group was 141, while only 39 in the BPE group. Phylum Firmicutes was the core taxonomy in MPE correlation network with 16 Firmicutes genera related with at least one genus.

Taxonomy structure composition did not differ significantly between the MPE and BPE groups. α diversity index between these two groups was similar (*p* = 0.3798 for chao1; *p* = 0.8148 for Simpson index; *p* = 0.8606 for Shannon) (Figure S2a‐c). β diversity, based on unweighted unifrac distance, did not reach significant results (ANOSIM test, *p* = 0.4129 for unweighted unifrac distance) (Figure S2a).

**FIGURE 2:**
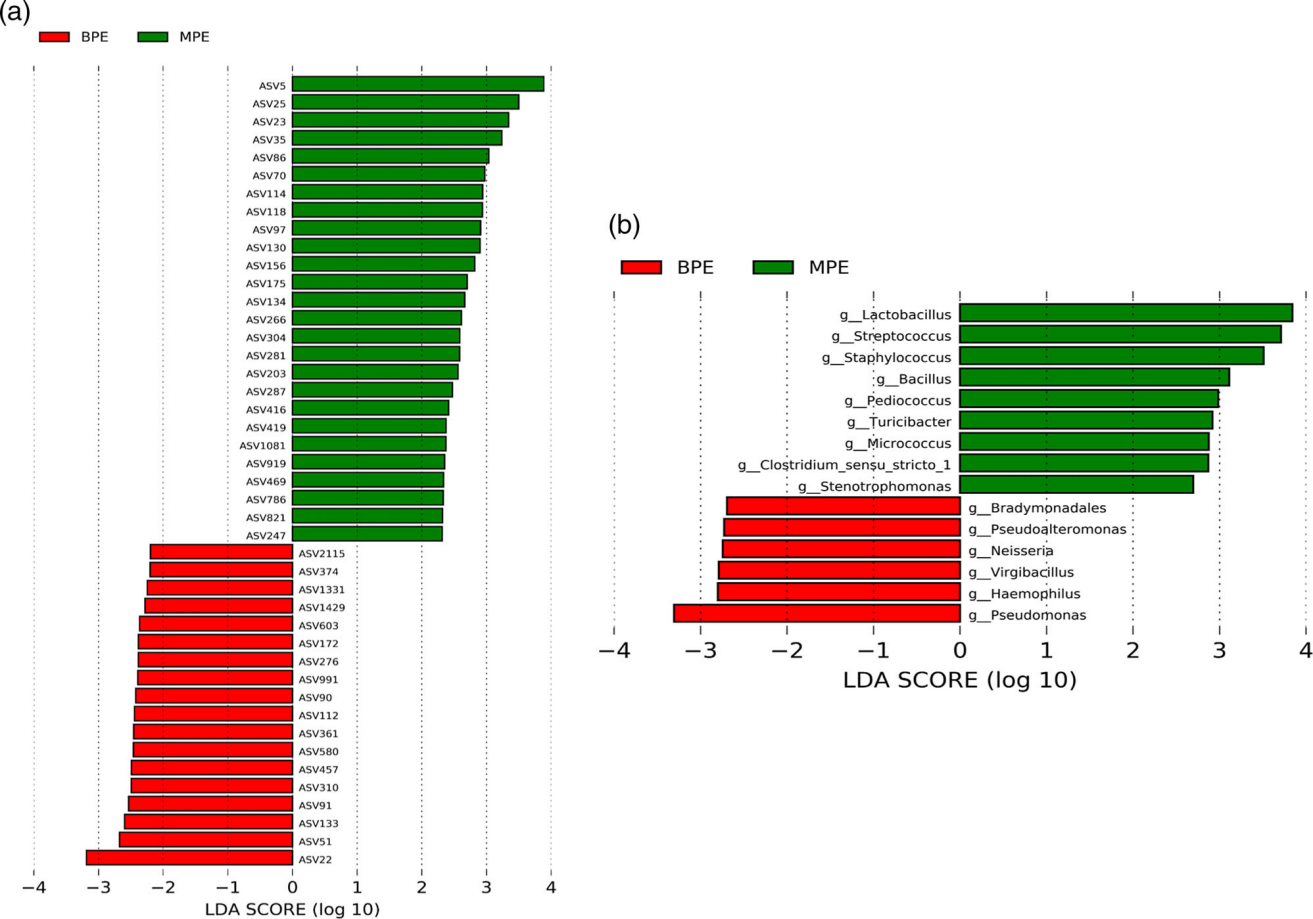
Differentially abundant pleural taxonomy identified by linear discriminant analysis effect size (LEfSe) analysis between malignant pleural effusion (MPE) and benign pleural effusion (BPE) individuals. (a) Differential amplicon sequence variants (ASVs) between the MPE and BPE groups. (b) Differential genera between the MPE and BPE groups.

MPE was associated with perturbed specific taxonomy abundance level. We identified 44 differential ASVs with relative abundance ≥0.1% between the MPE and BPE groups (Figure 2a). Among them, 26 ASVs were significantly enriched in the MPE group. Fourteen of them belonged to phylum *Firmicutes*, four belonged to genus *Lactobacillus*, and four ASVs could be annotated into species level (ASV 821: species *Corynebacterium_stationis*; ASV 266: species *Mycobacterium phlei*; ASV 416: species *Helicobacter_pylori*; ASV 35: species *Lactobacillus_delbrueckii*). Differential genera with relative abundance ≥0.1% are shown in Figure 2b. In detail, six differential genera including four *Proteobacteria* genera (*Neisseria*, *Pseudomonas*, *Haemophilus, Pseudoalteromonas*) were more increased in BPE samples while nine genera including seven *Firmicutes* genera (*Staphylococcus*, *Streptococcus*, *Lactobacillus*, *Bacillus*, *Turicibacter*, *Clostridium_sensu_stricto_1*, *Pediococcus*) were more increased in MPE patients. Details of the differential taxonomies are shown in Tables S2 and S3.

### Potential metabolic function alteration of MPE microbiota

**FIGURE 3:**
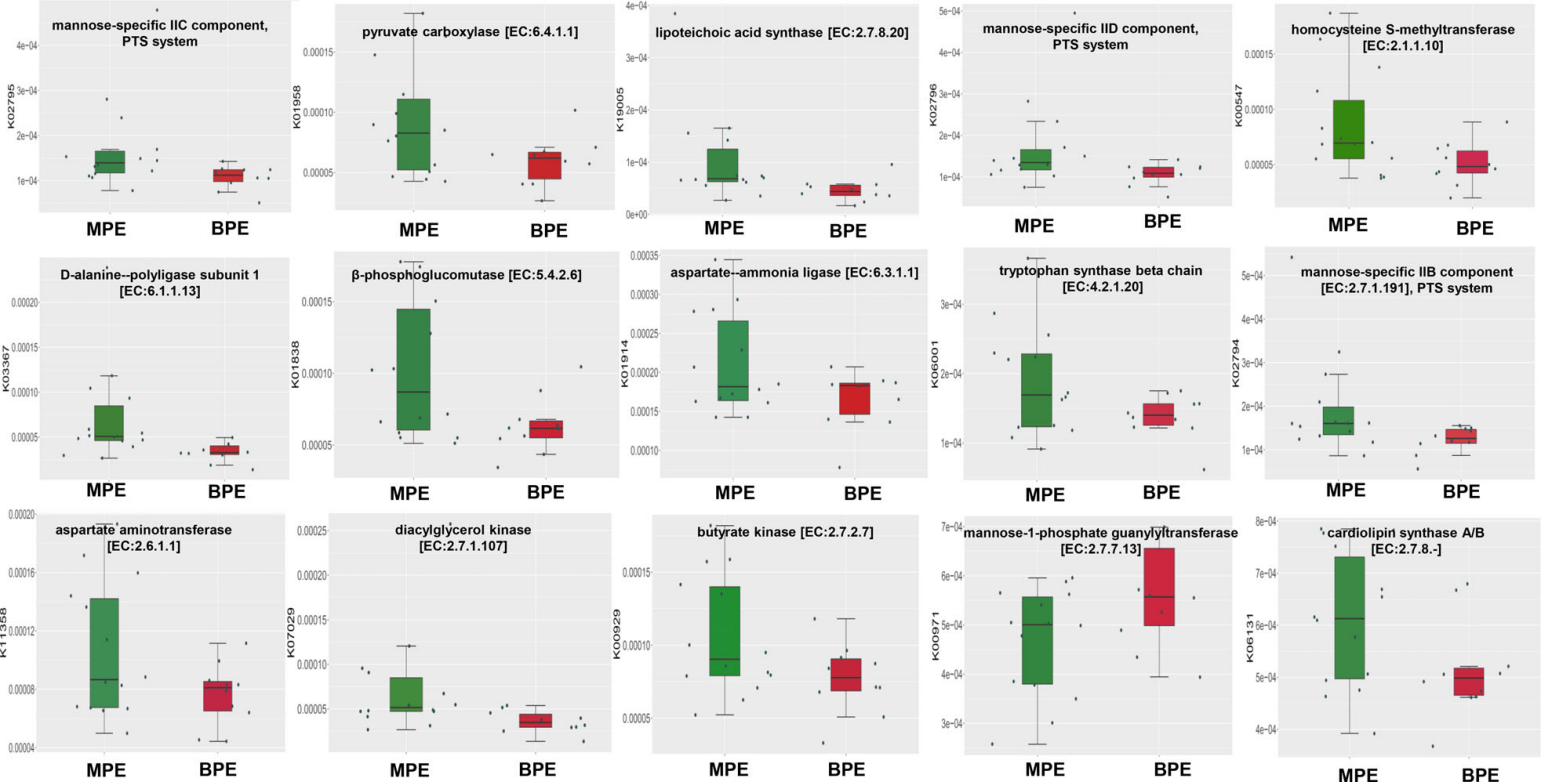
A total of 15 differential PICRUSTs predicted enzymes associated KO identified by Mann–Whitney U test.

To predict the possible impact of the altered pleural microbiome function in MPE in lung adenocarcinoma patients, ASVs were assigned to the closest reference genome in the database from the PICRUSt2 analysis. PICRUSt2 analysis showed that 114 significantly differential KEGG orthology (KO) between MPE and BPE. Fifteen of the KO could encode enzymes, which were involved with 42 specific KEGG metabolic pathways, such as carbohydrate metabolism (citrate cycle (TCA cycle); pyruvate metabolism), lipid biosynthesis and metabolism (glycerolipid metabolism; glycerophospholipid metabolism), amino acid metabolism (glycine, serine and threonine metabolism; phenylalanine, tyrosine and tryptophan biosynthesis; arginine biosynthesis; alanine, aspartate and glutamate metabolism). Fifteen differential KO which could encode enzymes are shown in Figure 3a. Further, a total of nine differential KEGG pathways were observed (Figure S3). Among them, two metabolic pathways including pyruvate and glycerolipid metabolism were significantly enriched in MPE patients.

### Pleural metabolic profile change associated with MPE in lung adenocarcinoma patients

To determine the alteration of pleural metabolites from MPE in lung adenocarcinoma patients, we conducted untargeted metabolomics through LC‐MS/MS. Molecules were analyzed using both positive and negative ionization. Pearson's correlation analysis between quality controls showed higher noise in the positive ion mode (Figure S4a, b). Therefore, a negative ion model was finally used. With coefficients of relative standard deviation <0.30 across QC samples, 457 metabolites were identified for further analysis. Among the 457 metabolites, eight metabolites were with abundance ≥100 million. These metabolites were LPC 16:0, elaidic acid, SM (d14:1/20:0), arachidonic acid, D‐(−)‐Fructose, LPC 18:0, docosahexaenoic acid and butylparaben.

**FIGURE 4:**
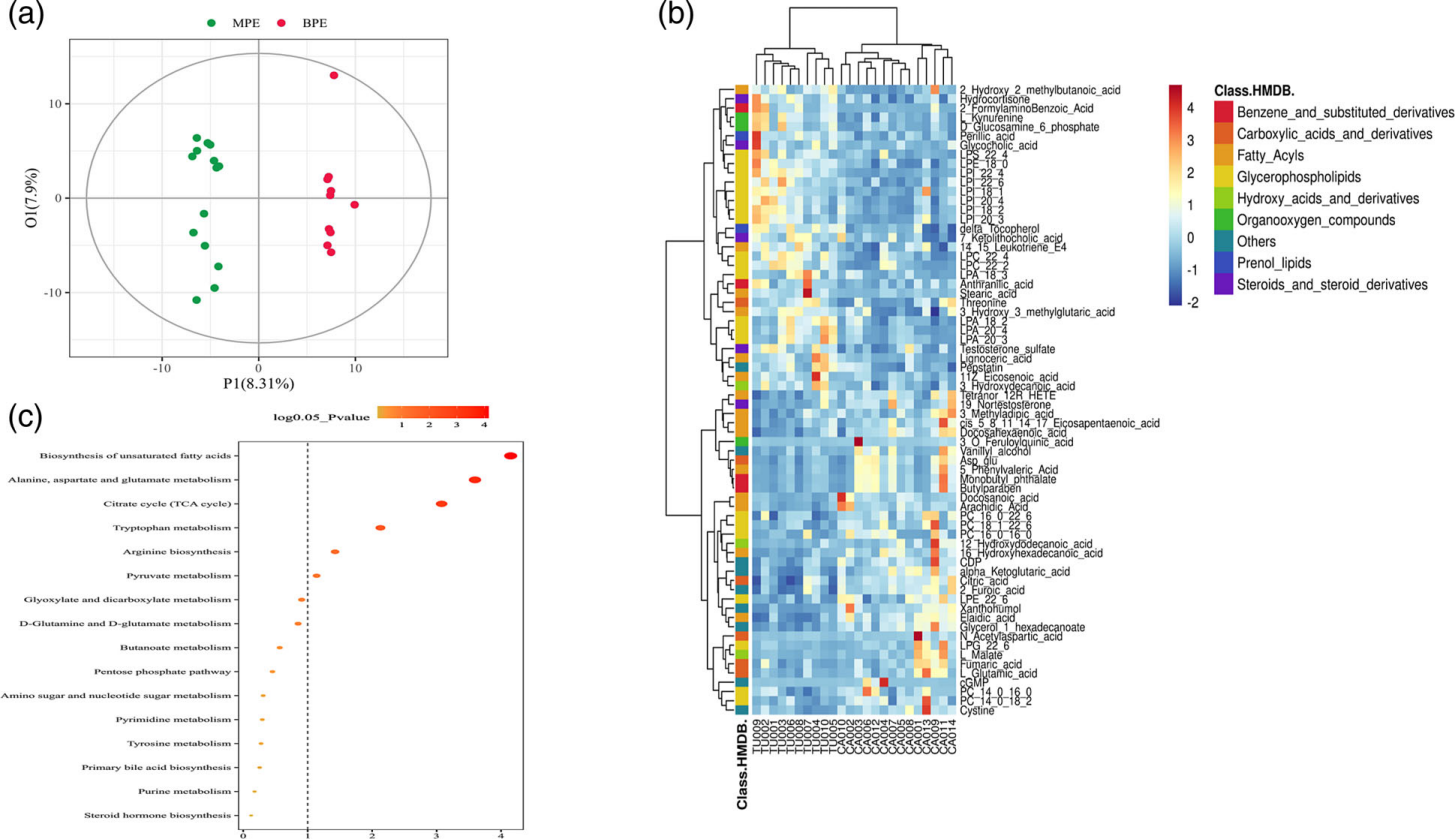
Screening and identification of the differential metabolites between malignant pleural effusion (MPE) and benign pleural effusion (BPE) groups. (a) The score plot of the metabolic differences between two groups by OPLS‐DA analysis. (b) Hierarchical clustering analysis for the MPE and BPE differential metabolites based on their *z*‐normalized abundances. (c) Bubble chart of differential enriched KEGG pathways between two groups. The size of the bubble is associated with the number of the differential metabolites implicated in the pathway.

In the OPLS‐DA model, metabolic profile of MPE were clearly distinguished from BPE, with cumulative R^2^Y = 0.995 and Q^2^Y = 0.807 (Figure 4a). A total of 143 metabolites were with VIP >1 and *p*‐value ≤0.05. Among the 143 metabolites, 121 of them with fold change ≥2 or ≤0.5 were selected as potential biomarkers of MPE and are listed in Table S4. Four metabolites with abundance ≥100 million (LPC 16:0, SM (d14:1/20:0), arachidonic acid, D‐(−)‐Fructose) of them showed higher abundance in MPE group but did not differ significantly. Among the 121 potential biomarkers, 68 differential metabolites could be annotated in HMDB or Lipid maps database. The variation tendencies of these 68 biomarkers were described by a hierarchical clustering analysis heat map in Figure 3b. These 68 differential metabolic biomarkers were mainly involved glycerophospholipids metabolites (lysophosphatidylinositols [LPI], lysophosphatidylcholine [LPC], lysophosphatidic acid [LPA], phosphatidylcholine [PC], etc.), fatty acyls (elaidic acid, 16‐hydroxyhexadecanoic acid, arachidic acid, docosanoic acid, etc.), carboxylic acids and derivatives (Asp‐glu, L‐glutamic acid, threonine, *N*‐acetylaspartic acid, citric acid, fumaric acid) and steroids and steroid derivatives (testosterone sulfate, hydrocortisone, 19‐nortestosterone, 7‐ketolithocholic acid, and glycocholic acid). An aberrantly changed lipid metabolite profile was observed. Among the differential glycerophospholipids metabolites, all LPI and LPC metabolites were significantly decreased in MPE and could be clustered together, while all PC metabolites were increased in MPE. The majority of differential fatty acyls metabolites (9 of 15) were significantly enriched in MPE and 3‐methyladipic acid, cis‐5,8,11,14,17‐eicosapentaenoic acid, docosahexaenoic acid, tetranor‐12(R)‐HETE, and 5‐phenylvaleric acid can be clustered together. Additionally, all carboxylic acids and derivatives except for threonine (citric acid, fumaric acid, L‐glutamic acid, Asp‐glu, *N*‐acetylaspartic acid) were significantly enriched in MPE.

Additionally, KEGG was applied and enriched pathways were identified based on differential metabolites. A total of 52 enriched pathways were obtained based on the significant differential metabolites, and six significantly enriched pathways (biosynthesis of unsaturated fatty acid, alanine, aspartate and glutamate metabolism, citrate cycle, tryptophan metabolism, arginine biosynthesis, pyruvate metabolism) possessed with most metabolites are shown in Figure 4c.

Spearman's analysis between 68 differential metabolites and four pleural fluid biochemical parameters were further observed (Figure S5). Pleural LDH was also significantly correlated with the greatest number of differential metabolites. Interestingly, LDH was positively related with differential metabolites associated with pyruvate metabolism (L‐malate and fumaric acid) and citrate cycle (L‐malate, fumaric acid and α‐ketoglutaric acid), reflecting the association of pleural tumor burden and energy consumption.

### Correlation between pleural metabolic phenotype and pleural microbiota

Based on the above pleural microbiome and metabolomic data, we performed Spearman's analysis to identify microbe‐associated in MPE. Interestingly, we observed that the microbe‐differential metabolite correlation was stronger in MPE patients than in BPE patients (data not shown), suggesting that the aberrantly differential metabolites in MPE group are partly attributed to pleural microbiota.

**FIGURE 5:**
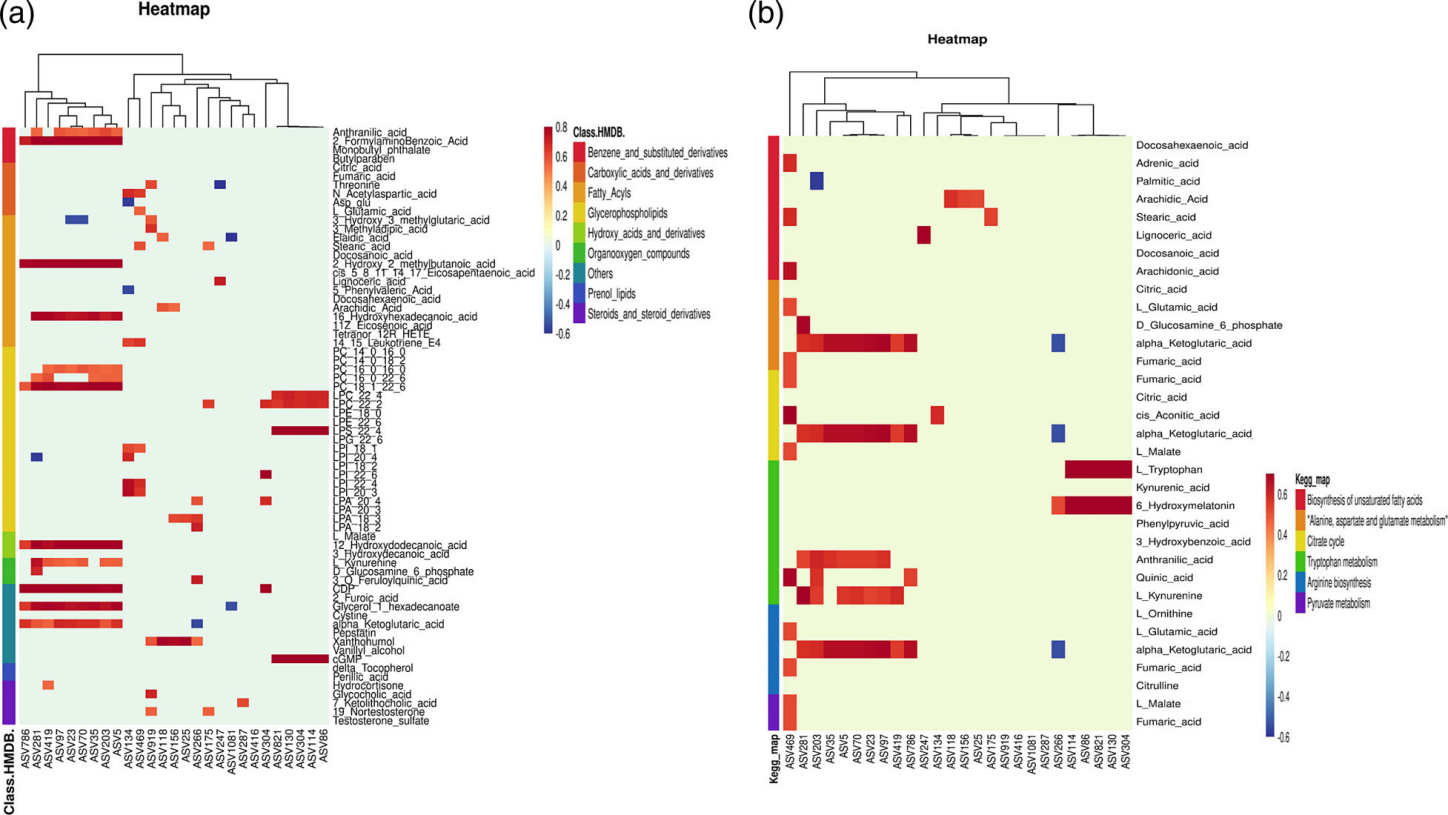
Spearman's correlation analysis (r) values for metabolites and malignant pleural effusion (MPE) enriched amplicon sequence variants (ASVs). Correlations with *p*‐values >0.05 were considered insignificant and numbered with 0. (a) Correlation heat map for differential metabolites among two groups and MPE enriched ASVs. (b) Correlation heatmap for metabolites implicated in differential enriched KEGG pathways for MPE and benign pleural effusion (BPE) groups and MPE enriched ASVs.

To further explore the correlation between MPE disturbed metabolites and MPE related enriched taxonomy, we formulated correlation matrix based on Spearman's correlation analysis. The results revealed several ASV‐metabolite pairs in the MPE group and PC (Figure 5a, Table S5), particularly involved with almost all PC and all LPC metabolites. For example, several PC metabolites (PC (16:0/16:0), PC (16:0/22:6), PC (18:1/22:6)) were positively associated with ASV 35 (species *Lactobacillus_delbrueckii*). At the genus level, several PC metabolites were associated with 6 *Firmicutes* genera, included *Streptococcus*, *Lactobacillus*, *Bacillus*, *Turicibacter*, *Clostridium_sensu_stricto_1*, *Pediococcus* (Figure S6, Table S6). The results suggested that the aberrantly dysregulated metabolites in MPE were partly attributed to an imbalance pleural microflora or to interactions thereof.

As mentioned above (Figure 4b), MPE was significantly enriched in six KEGG metabolic pathways. Correlation analysis was conducted between the metabolites involved with these six pathways and the MPE enriched ASVs (Figure 5b) (Table S7). Several metabolites which participate in the six KEGG pathways were associated with specific ASVs. Particularly, almost all metabolites involved with citrate cycle (fumaric acid, citric acid, cis‐aconitic acid, α‐ketoglutaric acid, L‐malate) and all metabolites involved with pyruvate metabolism (fumaric acid, L‐malate) were associated with MPE enriched ASVs. For example, fumaric acid, cis‐aconitic acid and L‐malate were associated with ASV469. Genes related to the production of fumaric acid, include gene K01677 (encoding enzyme fumarate hydratase subunit alpha), gene K01678 (encoding enzyme fumarate hydratase subunit beta), gene K00244 (encoding succinate dehydrogenase flavoprotein subunit), gene K00245 (encoding succinate dehydrogenase iron–sulfur subunit), and gene K00246 (encoding succinate dehydrogenase subunit C) were found in the closet genome of ASV469. K01677 and K01678 were also responsible for encoding enzymes that catalyze fumaric acid into L‐malate. α‐ketoglutaric acid was associated with most MPE enriched ASVs (ASV203, ASV23, ASV266, ASV281, ASV35, ASV419, ASV5, ASV70, ASV786, ASV97). Genes related to the production of α‐ketoglutaric acid included K00031 and K00260. Gene K00031 encoding isocitrate dehydrogenase that can catalyze isocitrate into α‐ketoglutaric acid was found in the closet genome of ASV5, ASV 266, ASV786. Gene K00260 encoding glutamate dehydrogenase that catalyzes L‐glutamate into α‐ketoglutaric acid was found in the closet genome of ASV419, ASV786.

## DISCUSSION

In this study, we collected pleural samples from lung adenocarcinoma and tuberculosis patients and used an integrated 16S rRNA gene sequencing and LC‐MS‐based metabolomic approach to explore the characterization of microbiota and metabolomic profile of MPE. Our results demonstrated several significantly enriched genera and ASVs in the MPE microbial community; meanwhile, the host metabolic profile was also perturbed in MPE samples. Furthermore, the results showed a significant association between perturbed MPE taxonomies and pleural metabolites, suggesting that the aberrantly dysregulated metabolites in MPE were attributed to an imbalance pleural microflora, or to interactions thereof.

Using an untargeted approach, we identified several differential metabolites between MPE and BPE samples. The majority of the differential metabolites were lipid, which included glycerophospholipids and fatty acids. Lipids are involved in various cell processes, including energy supply, cell membrane constitution, and serve as messengers in signal transduction and molecular recognition processes.^20^ Increasing evidence has shown that lipids play an important role in the occurrence and development of lung cancer.^21^ In our study, we found that multiple LPC and LPI metabolites were significantly decreased in MPE, while all PC metabolites were increased in MPE. LPCs have been identified as a group of proinflammatory lipids that are involved in the pathogenesis of multiple inflammatory diseases.^22^, ^23^ However, their role in lung cancer remains uncertain. Consistent with our study, Yang et al. also found several LPC metabolites were significantly decreased in MPE.^4^ Under physiological conditions, LPC is cleared by enzymes such as acyl‐CoA:LPCAT.^24^ Multiple studies have demonstrated that overexpression of the LPCAT gene may contribute to the progression and metastasis of human cancer, including lung cancer.^22^ A recent study demonstrated that the LPCAT gene was upregulated in a lung adenocarcinoma tissue and cell line, and LPCAT at least partially influenced lung adenocarcinoma metastasis through the PI3K/AKT signal pathway by targeting MYC transcription.^25^ Thus, it seems reasonable that the low LPC level in MPE might be regulated by the LPCAT activity. PC are the most abundant surfactants lipids and play an importantly protective role in oxidation stress.^26^ In our study, PC was found to be significantly increased in MPE, which contradicted with the results of Yang et al.^4^ The inconsistency between both studies might be attributed to the heterogeneity of lung cancer, the difference of the participants enrolled and study techniques. A recent study using lipidomics found that PC was elevated in early‐stage lung cancer and could serve as biomarkers for the early detection of lung cancer.^27^ Another recent study found that PC was elevated in a K‐ras mutated lung adenocarcinoma mice model and was indicative of oxidative stress in lung cancer.^28^ Therefore, the role of PC in lung cancer at different stages needs further explanation. Apart from aberrant lipid metabolism in MPE, we also found several amino acids (L‐glutamic acid, Asp‐glu, *N*‐acetylaspartic acid) were elevated in MPE patients. Dysregulated metabolism of amino acids, especially glutamine, serine and glycine, have been identified to function as important regulators in supporting cancer cell growth.^29^ A recent case–control study in a Chinese population found that serum glutamic acid and Asp‐Glu were found to be elevated in lung cancer patients, especially those in late stage disease.^30^ In general, our global nontargeted metabolomic efforts in pleural samples from MPE and BE individuals showed multiple differential metabolic signatures, which pend further validation studies.

Among the eight metabolites with abundance ≥100 million, four (LPC 16:0, SM [d14:1/20:0], arachidonic acid, D‐[−]‐fructose) of them showed higher abundance in the MPE group but did not differ significantly. It is worthwhile to discuss their role in MPE, owing to the small sample size of our study. SM (d14:1/20:0) belonged to sphingomyelin. Increased sphingomyelin abundance has been reported to play a critical role in cell proliferation and survival in several cancer types.^31^, ^32^ Arachidonic acid is an n‐6 essential fatty acid and is constituted of biomembranes. Several studies have reported that arachidonic acid‐derived lipid mediators can regulate cancer initiation and progression,^33^, ^34^ including lung cancer. A recent study also reported that increased blood level of arachidonic acid was found to be associated with higher lung cancer risk.^35^

Perturbed microbiota may aggravate cancer development and progression through multiple mechanisms.^36^, ^37^ The microbiome inhabits several human organs and cavities. Multiple studies have demonstrated a bacterial community exists in ascetic fluid which is perturbed under several diseases such as liver cirrhosis.^38^, ^39^, ^40^ However, the composition of microbiota in pleural effusion and its function remains largely unknown. In our study, using 16S rRNA gene sequencing, we found phyla *Firmicutes*, *Bacteroidetes*, and *Proteobacteria* were dominant members of the microbiota community in both MPE and BPE groups. Further, we found nine genera including seven *Firmicutes* genera (*Staphylococcus*, *Streptococcus*, *Lactobacillus*, *Bacillus*, *Turicibacter*, *Clostridium_sensu_stricto_1*, *Pediococcus*) were significantly enriched in MPE individuals. Lung and gut *Firmicutes* have been found to be a useful microbial signature lung cancer in several studies.^41^, ^42^, ^43^ Several *Firmicutes* members including genus *Streptococcus*,^44^, ^45^, ^46^ *Staphylococcus*,^44^ *Lactobacillus*,^47^, ^48^ and *Pediococcus* ^48^ have recently been implicated in lung cancer patients. The role of pleural microbiota needed further investigation and it is plausible that the tumor‐promoting effects of the microbiome in MPE may probably be caused by holistic dysbiosis, rather than by a specific pathogen.

Accumulating data suggests that metabolic phenotype changes are correlated with microbiota perturbations in many diseases, including obesity, type 2 diabetes, nonalcoholic liver disease, cardio‐metabolic diseases and malnutrition.^49^, ^50^ In this study, we observed a significant correlation between MPE disturbed metabolites and MPE‐related enriched taxonomies. For example, several enriched PC metabolites have been associated with ASV 35 (species *Lactobacillus_delbrueckii*). *Lactobacillus_delbrueckii*, as a *lactobacillus* strain, has been previously reported to have a therapeutic effect in cancer.^51^, ^52^ Supplementation of *Lactobacillus_delbrueckii* might be tightly linked to host lipid metabolism via colonic microbiota modulation.^53^ Thus, it seems reasonable that the enriched *Lactobacillus_delbrueckii* in MPE is capable of producing more PC, which is indicative of an intense oxidative stress in MPE individuals. KEGG pathway enrichment analysis has shown that citrate cycle and pyruvate metabolism are upregulated in MPE, which might reflect increased energy consumption in the cancer environment. Interestingly, almost all metabolites involved with the citrate cycle and all metabolites involved with pyruvate metabolism are associated with MPE enriched ASVs. For instance, α‐ketoglutaric acid was found to be associated with most MPE enriched ASVs. Several studies have shown that gut microbiota is associated with host citrate cycle^54^, ^55^, ^56^ and pyruvate metabolism in cancer.^57^ Several metabolites that are involved in citrate cycle process might regulate tumor progression.^58^ A recent study demonstrated that the α‐ketoglutaric acid is an important metabolite that can regulate P53‐mediated tumor suppression effect.^59^ Acclamations of fumaric acid elicited an epithelial‐to‐mesenchymal‐transition in tumor cells and promote tumor progression.^60^ Thus, it might be plausible that MPE microbiota might affect pleural metastasize of lung adenocarcinoma through modulation of certain metabolites.

There are some limitations in our study. First, the number of patients enrolled in this study was not large enough, so there may be heterogeneity. Second, it is challenging to evaluate pleural metabolites and to differentiate host‐derived metabolites from those of the microbial metabolites. Third, the study was cross‐sectional and only illustrates the microbiome‐metabolome associations phenomenon at one time point. The mechanism of the microbiota and the causal relationship need further exploration. Despite the limitations, it opens a new gate to explore the pleural microbiome‐metabolome associations in MPE patients for biomarker discovery.

In conclusion, in this study, we combined untargeted LC/MS analysis and 16S rRNA analysis to assess the characterization of metabolic profile and microbiota community in MPE. The results showed that the metabolic profile of MPE was clearly distinguished from BPE, with several differential metabolites including glycerophospholipids, fatty acids, etc. enriched in MPE. Furthermore, pleural microbiota analysis showed signature taxonomies represented the MPE, that is, enrichment of several *Firmicutes* genera, such as *Streptococcus*. Additionally, correlation analysis revealed that some altered pleural microbiota genera and ASVs were strongly correlated with perturbed metabolites. This integrated analysis of the putative microbial metabolism based on the microbes and pleural metabolites provides more functional insights than either of the single datasets.

## AUTHOR CONTRIBUTIONS

Conceptualization: Hangming Dong, Shaoxi Cai, DanHui Huang. Formal analysis: DanHui Huang, JinZhong Zhuo, CuiPing Ye; Data curation: JinZhong Zhuo, CuiPing Ye, XiaoFang Su, YueHua Chen, QianNan Ren; Project administration: JinZhong Zhuo, CuiPing Ye, XiaoFang Su, YueHua Chen, Cui Li, LiSan Lin, LaiYu Liu, Haijin Zhao, Tingyue Luo JianHua Wu; Funding acquisition: DanHui Huang, Shaoxi Cai, Hangming Dong; Writing‐original draft: DanHui Huang, JinZhong Zhuo, CuiPing Ye; Writing‐review and editing: Shaoxi Cai, Hangming Dong.

## CONFLICT OF INTEREST STATEMENT

The authors declare no conflict of interests.

## Supporting information


**Figure S1.** ASV rarefaction curve of MPE individuals and BPE individuals based on Simpson analysis.
**Figure S2.** Diversity of pleural microbiota for MPE and BPE groups. (a) Shannon index; (b) Chao1 index; (c) Simpson index; (d) Shannon index; (e) PCOA plot based on unweighted unifrac distance.
**Figure S3.** Differential PICRUST2 predicted KEGG pathways identified by LEfSe analysis for pleural microbiota of MPE and BPE groups.
**Figure S4.** Pearson correlation matrix between (a) quality controls (QCs) in positive ion model and (b) QCs in negative ion model.
**Figure S5.** Spearman's correlation (r) values for differential metabolites among 2 groups and pleural effusion parameters. Correlations with *p*‐value >0.05 were considered insignificant and numbered with 0.
**Figure S6.** Spearman correlation (r) values for differential metabolites among 2 groups and MPE enriched genera.

**Table S1.** The clinicopathological factors for the MPE and BPE patients.
**Table S2.** Differential ASVs identified by LEfSe between MPE and BPE group.
**Table S3.** Differential genera identified by LEfSe between MPE and BPE group.
**Table S4.** Differential metabolites between MPE and BPE patients.
**Table S5.** Spearman correlation (r) values for differential metaolites and MPE enriched ASVs.
**Table S6.** Spearman's correlation (r) values for differential metaolites and MPE enriched genera.
**Table S7.** Spearman correlation (r) values for metaolites involved in KEGG enriched pathways and MPE enriched ASVs.
