# Supplementary material for: Variations in pleural microbiota and metabolic phenotype associated with malignant pleural effusion in human lung adenocarcinoma
Source: Thorac Cancer. 2023 Jun 12;14(21):2045–56. doi: 10.1111/1759-7714.14988 (PMC10363824; doi:10.1111/1759-7714.14988)
Supplement: Supplementary file 1 — Figure S1. ASV rarefaction curve of MPE individuals and BPE individuals based on Simpson analysis. Figure S2. Diversity of pleural microbiota for MPE and BPE groups. (a) Shannon index; (b) Chao1 index; (c) Simpson index; (d) Shannon index; (e) PCOA plot based on unweighted unifrac distance. Figure S3. Differential PICRUST2 predicted KEGG pathways identified by LEfSe analysis for pleural microbiota of MPE and BPE groups. Figure S4. Pearson correlation matrix between (a) quality controls (QCs) in positive ion model and (b) QCs in negative ion model. Figure S5. Spearman's correlation (r) values for differential metabolites among 2 groups and pleural effusion parameters. Correlations with p‐value >0.05 were considered insignificant and numbered with 0. Figure S6. Spearman correlation (r) values for differential metabolites among 2 groups and MPE enriched genera. [file TCA-14-2045-s002.pdf]

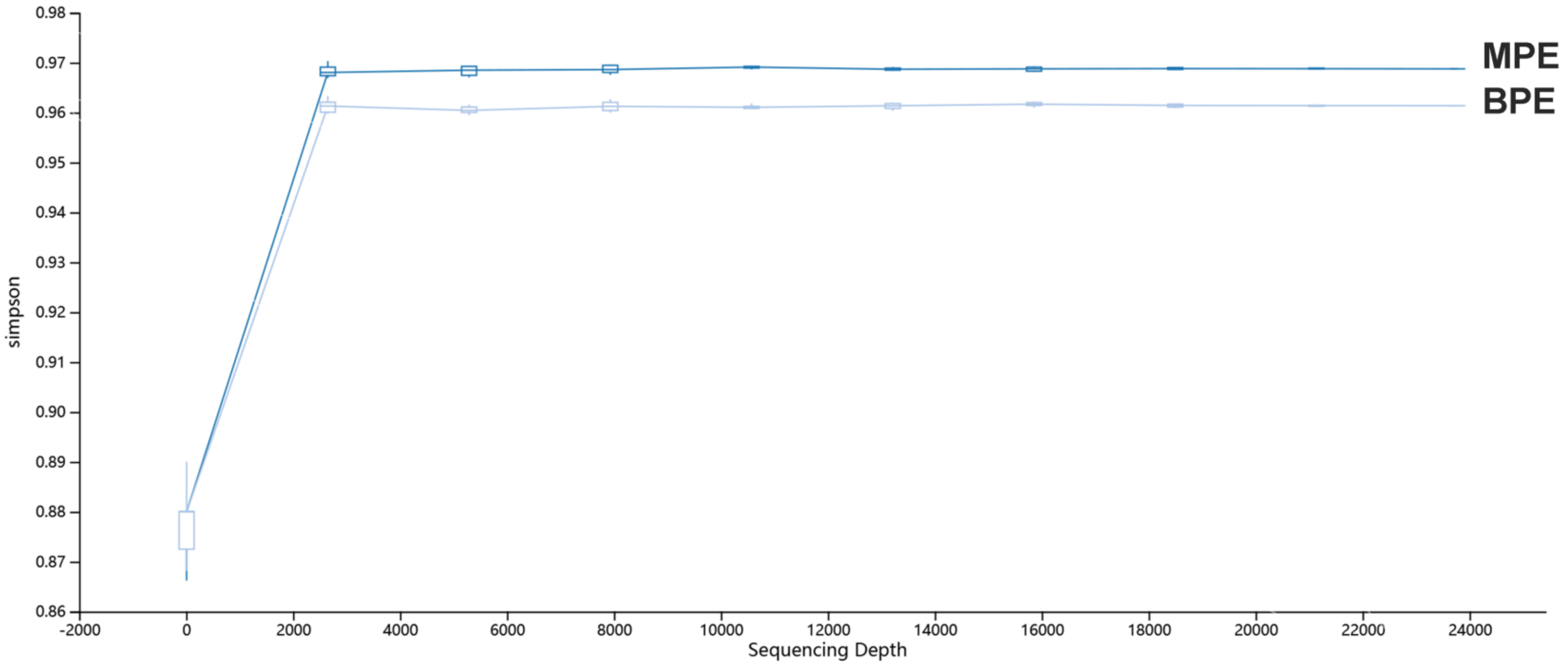

Supplementary Figure S1

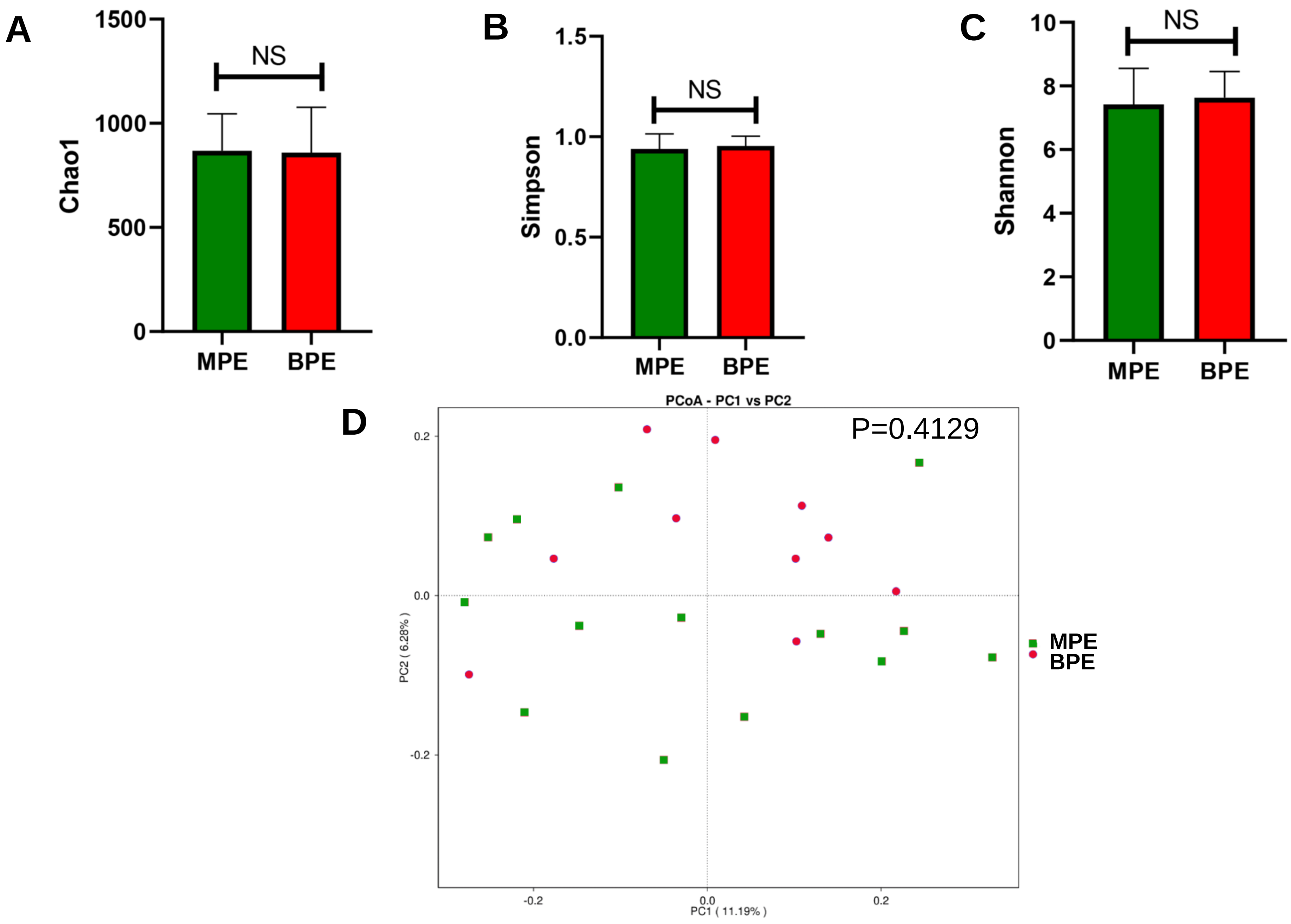

Supplementary Figure S2

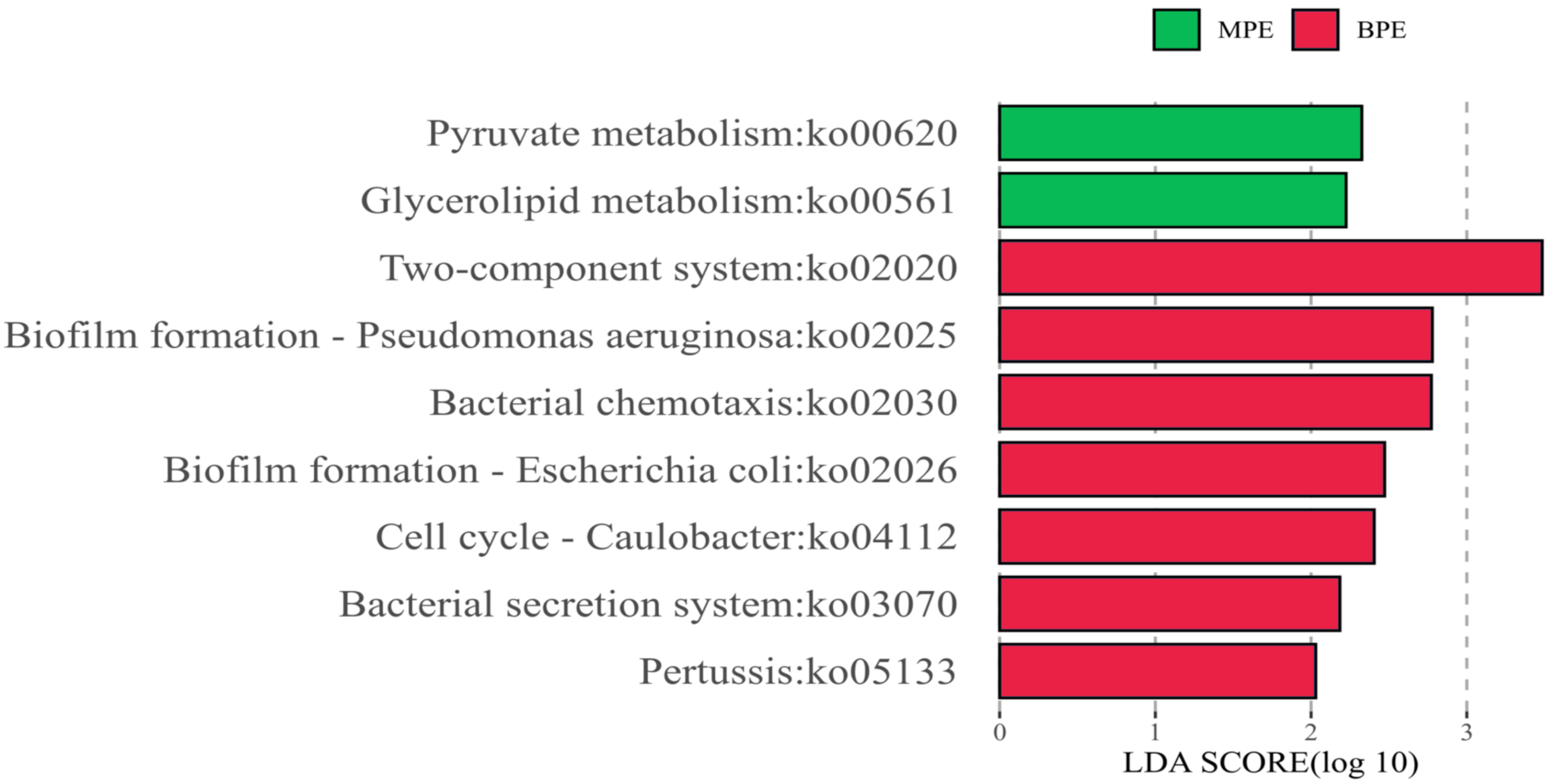

Supplementary Figure S3

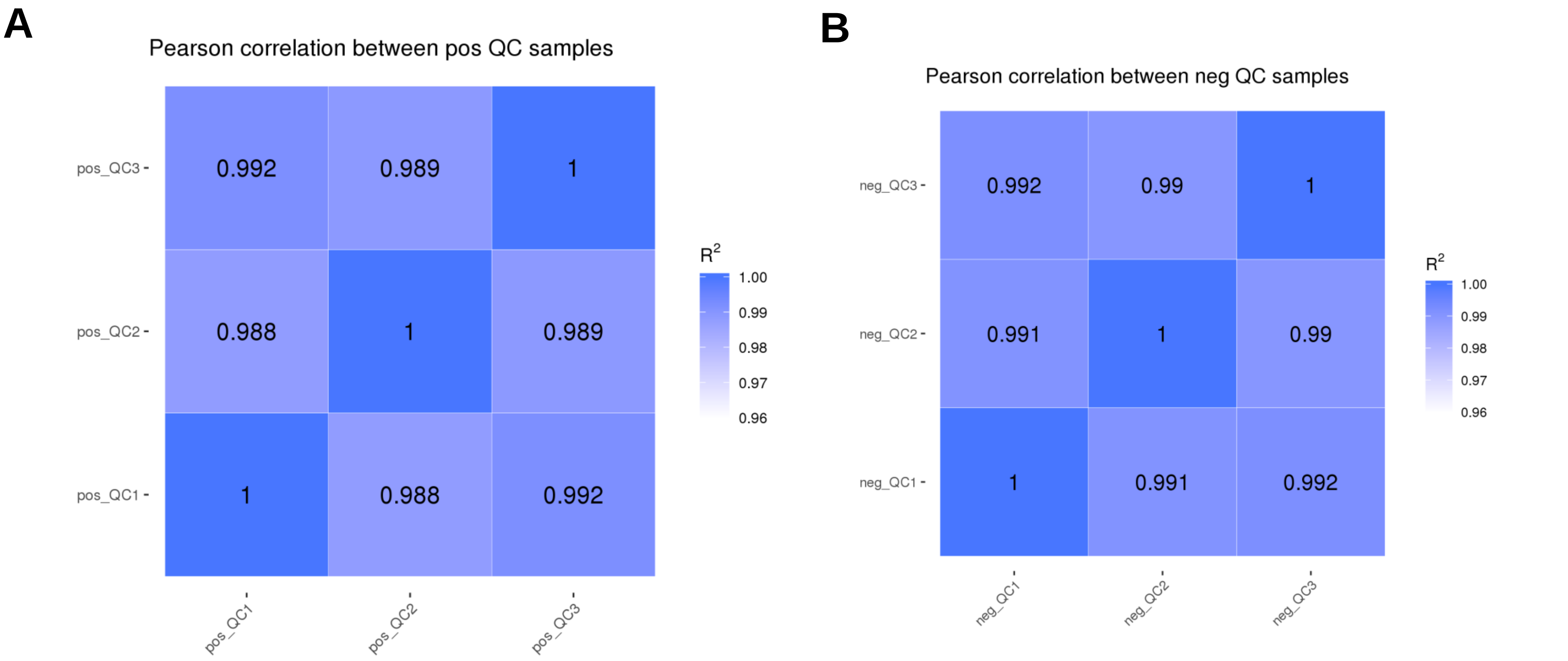

Supplementary Figure S4

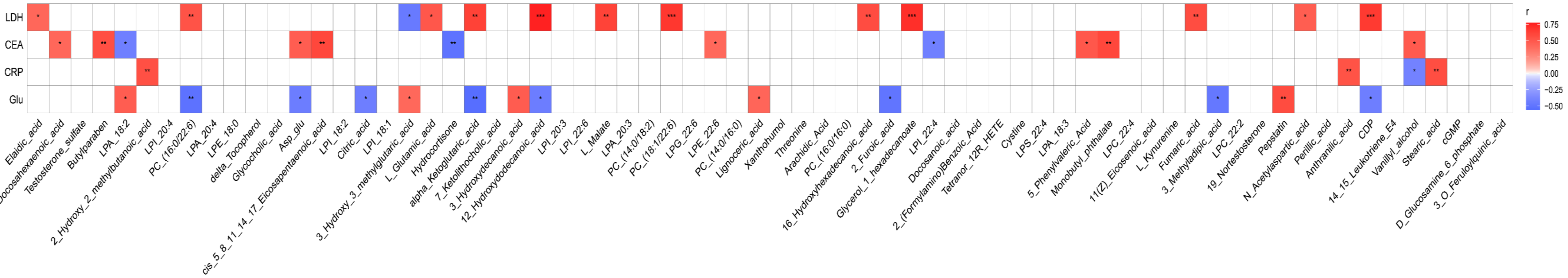

Supplementary Figure S5

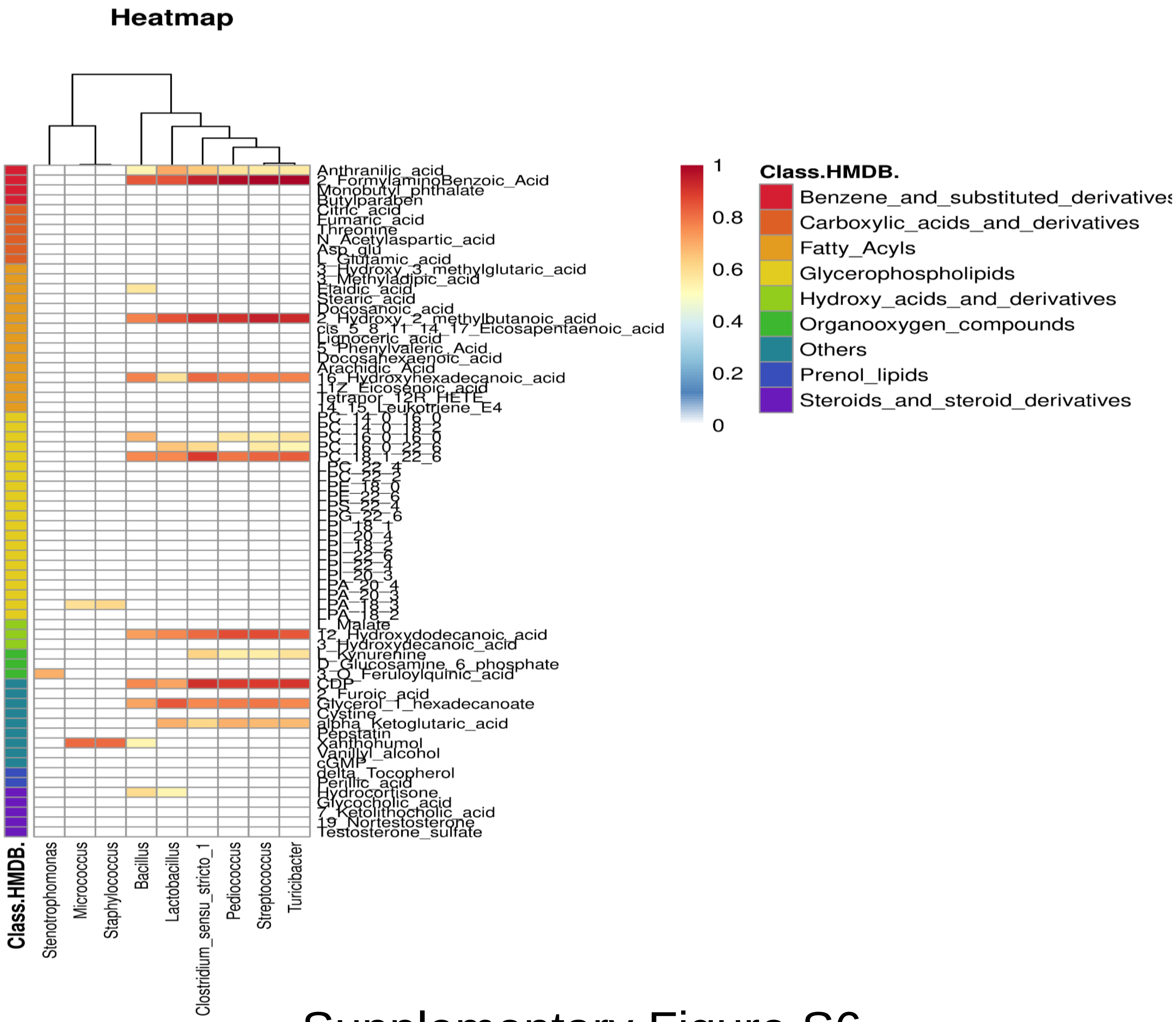

Supplementary Figure S6
